# Efficacy of Goshajinkigan for Oxaliplatin-Induced Peripheral Neuropathy in Colorectal Cancer Patients

**DOI:** 10.1155/2013/139740

**Published:** 2013-11-07

**Authors:** Naohisa Yoshida, Toyoshi Hosokawa, Takeshi Ishikawa, Nobuaki Yagi, Satoshi Kokura, Yuji Naito, Masayoshi Nakanishi, Yukihito Kokuba, Eigo Otsuji, Haruo Kuroboshi, Masafumi Taniwaki, Tetsuya Taguchi, Hajime Hosoi, Terukazu Nakamura, Tsuneharu Miki

**Affiliations:** ^1^Department of Molecular Gastroenterology and Hepatology, Graduate School of Medical Science, Kyoto Prefectural University of Medicine, 465 Kajii-cho, Kawaramachi-Hirokoji, Kamigyo-ku, Kyoto 602-8566, Japan; ^2^Outpatient Oncology Unit, Kyoto Prefectural University of Medicine Hospital, Kyoto 602-8566, Japan; ^3^Pain Treatment and Palliative Care Unit, Kyoto Prefectural University of Medicine, Kyoto 602-8566, Japan; ^4^Division of Digestive Surgery, Graduate School of Medical Science, Kyoto Prefectural University of Medicine, Kyoto 602-8566, Japan; ^5^Department of Obstetrics and Gynecology, Kyoto Prefectural University of Medicine, Kyoto 602-8566, Japan; ^6^Division of Hematology and Oncology, Graduate School of Medicine, Kyoto Prefectural University of Medicine, Kyoto 602-8566, Japan; ^7^Division of Endocrinological and Breast Surgery, Kyoto Prefectural University of Medicine, Kyoto 602-8566, Japan; ^8^Department of Pediatrics, Graduate School of Medical Science, Kyoto Prefectural University of Medicine, Kyoto 602-8566, Japan; ^9^Department of Urology, Graduate School of Medicine, Kyoto Prefectural University of Medicine, Kyoto 602-8566, Japan

## Abstract

*Objective*. To evaluate the efficacy of Goshajinkigan for oxaliplatin-induced peripheral neuropathy in colorectal cancer patients. *Patients*. Colorectal cancer patients (*N* = 29) who received ≥4 weeks of Goshajinkigan for oxaliplatin-induced peripheral neuropathy during chemotherapy at Kyoto Prefectural University of Medicine were (Goshajinkigan group) compared to 44 patients who had not received Goshajinkigan during the same period (non-Goshajinkigan group). *Main Outcome Measures*. The effect of Goshajinkigan was graded as curative, effective, stabilizing, or deleterious. The relationships between the grade of peripheral neuropathy and the dose of oxaliplatin in the Goshajinkigan and non-Goshajinkigan groups were evaluated. *Results*. The effect of Goshajinkigan on peripheral neuropathy in the Goshajinkigan group was curative, effective, stabilizing, and deleterious in 3.4, 20.7, 69.0, and 6.9% of patients, compared to the effect in the non-Goshajinkigan group (4.5, 15.9, 45.5, and 34.1%). The ratio of deleterious effects was significantly different between these two groups (*P* = 0.04). A Kaplan-Meier analysis in relation to the cumulative dose of oxaliplatin showed that the incidence of grade 3 peripheral neuropathy tended to be less in the Goshajinkigan group (*P* = 0.05). There were no significant differences in time to treatment failure and severe adverse events between these two groups. *Conclusions*. Goshajinkigan prevented exacerbation of oxaliplatin-induced peripheral neuropathy. This trial is registered with UMIN000009956

## 1. Introduction

Oxaliplatin-based chemotherapy regimens such as XELOX (CapeOX) and FOLFOX have gained acceptance worldwide as first-line therapies in advanced or recurrent colorectal cancer; subsequently, the incidence of the often intractable oxaliplatin-induced peripheral neuropathy also continues to rapidly increase [1]. Peripheral neuropathy occurs in approximately 80% of the patients receiving oxaliplatin-based chemotherapy. Grade 3 peripheral neuropathy affects approximately 15% of the patients after a cumulative dose of 800 mg/m^2^ and requires discontinuation of therapy [2]. The unique direct effect of oxaliplatin on nerve excitability has been attributed to one of its metabolites, oxalate, which is a calcium chelator that alters voltage-gated Na^+^ channels. In experimental models, acute dysfunction of Na^+^ channels is correlated with axonal loss and degeneration [3]. Furthermore, cumulative neurotoxicity may be related to direct toxicity of the dorsal root ganglia [4]. The hallmarks of oxaliplatin-induced peripheral neuropathy are paresthesia and dysesthesia in the distal extremities or a peculiar sensory deficit that is triggered or aggravated by exposure to cold [5]. Although a new Japanese guideline for the management of neuropathic pain was established in 2011 that advocates the use of antidepressants, pregabalin, and tramadol for this condition, no treatment protocol for oxaliplatin-induced peripheral neuropathy has been firmly established to date [6–8]. In Japan, Kampo medicines such as Goshajinkigan (GJG) that have been approved by the Ministry of Health, Labor, and Welfare as pharmaceutical drugs are used for the management of peripheral neuropathy. GJG is a mixture of extracts from 10 botanical raw materials in fixed proportions. The main indications for GJG are leg pain, lower back pain, and diabetic neuropathy [9]. Two mechanisms have been postulated to explain the ability of GJG to alleviate peripheral neurotoxicity. First, GJG may stimulate spinal dynorphin release, which would robustly activate *κ*-opioid receptors and block pain transmitters. Second, GJG may enhance nitric oxide production and thereby increase blood circulation while inhibiting blood coagulation. Taken together, these molecular events suggest enhanced blood supply to the nerve and the activation of an endogenous pain modulating system [10, 11]. Moreover, a recent report by Ushio et al. showed that GJG relieves oxaliplatin-induced cold hyperalgesia and mechanical allodynia without affecting the antitumor activity of oxaliplatin in rats [12]. Only a few studies indicated that GJG is effective against oxaliplatin-induced peripheral neuropathy in colorectal cancer patients [13–16]. In the current study, we evaluated the efficacy of GJG for the treatment of oxaliplatin-induced peripheral neuropathy.

## 2. Patients and Methods

Twenty-nine evaluable colorectal cancer patients (17 men and 12 women; mean age 60.4 years) who were treated with GJG (Tsumura & Co., Tokyo, Japan) for peripheral neuropathy due to oxaliplatin-based chemotherapy at the Outpatient Oncology Unit of Kyoto Prefectural University of Medicine Hospital between April 2009 and August 2012 were identified retrospectively for the study (the GJG group). Patients who did not receive GJG for peripheral neuropathy during the same period were analyzed for comparison (the non-GJG group). This study was approved by the ethics committee of Kyoto Prefectural University of Medicine and was carried out in accordance with the World Medical Association Helsinki Declaration (adopted in 1964 and amended in 1975, 1983, 1989, 1996, 2000, 2002, 2004, and 2008). This study has been registered in the University Hospital Medical Information Network Clinical Trials Registry (UMIN-CTR) as number UMIN000009956.

 Inclusion criteria were restricted to colorectal cancer patients receiving GJG for peripheral neuropathy related to oxaliplatin-based chemotherapy. The regimen of oxaliplatin-based chemotherapy was defined as XELOX or FOLFOX. Patients administered molecular target drugs such as cetuximab, bevacizumab, or panitumumab with oxaliplatin-based chemotherapy were also included. Patients receiving more than three courses of these regimens such as XELOX and FOLFOX were included. Patients were assigned to the GJG and non-GJG groups by the clinical doctor. Additionally, we included patients receiving GJG from both the beginning of chemotherapy (for prevention of peripheral neuropathy) and the middle of chemotherapy (to treat peripheral neuropathy). Clinical doctors decided whether GJG was to be prescribed from the beginning of chemotherapy. On the other hand, all patients who did not receive GJG during the same period were included in the non-GJG group. Exclusion criteria were patients with severe organ disorders and patients receiving other treatments such as pregabalin, tramadol, opioids, calcium gluconate, and magnesium sulfate for peripheral neuropathy. Moreover, patients administered GJG for less than three weeks were excluded.

 The following patient characteristics were analyzed: age, sex, activity level, cancer stage, chemotherapy regimen, and cumulative dose of oxaliplatin. Cancer stages were classified according to UICC-TNM [17]. The ECOG scale for performance status (PS) was used for evaluating the patients' activity [18]. The severity of peripheral neuropathy was graded according to the CTCAE v. 4.0: grade 0, asymptomatic; grade 1, mild sensory alteration or paresthesia (not requiring intervention); grade 2, sensory alteration or paresthesia that moderately interferes with instrumental activities of daily living (ADLs); grade 3, sensory alteration or paresthesia that severely interferes with basic ADLs; and grade 4, disabling neuropathy [19]. Instrumental ADLs consisted of tasks related to independent living such as preparing meals, shopping for groceries or personal items, using a telephone, and taking medications. Basic ADLs consisted of self-care tasks such as bathing, dressing, eating, excretion, and ambulation. Analyses of the grade of peripheral neuropathy and treatment effects of GJG were based on patient paper interviews with helps of outpatient-center nurses. The effect of GJG for peripheral neuropathy was evaluated at the end of oxaliplatin-based chemotherapy and was graded as curative, effective, stabilizing, or deleterious. In detail, the efficacy of GJG was evaluated based on whether it was administered from the beginning of chemotherapy or from the middle of chemotherapy. For the patients receiving GJG from the beginning of chemotherapy for prevention of peripheral neuropathy, the effect of GJG was evaluated as follows: if the symptoms reached grade 0, 1, 2, or 3/4 by the end of treatment, the effect was defined as curative, effective, stabilizing, or deleterious, respectively. For patients receiving GJG from the middle of chemotherapy for the treatment of peripheral neuropathy, the effect of GJG was evaluated as follows: when the symptoms had improved completely, the effect was defined as curative; when the symptoms improved by one grade (CTCAE), the effect was defined as effective; when the symptoms stabilized, the effect was defined as stabilizing; and when the symptoms worsened by one grade, the effect was defined as deleterious. 

 A Kaplan-Meier analysis of grade 3 peripheral neuropathy in relation to total oxaliplatin dose was performed in the GJG and non-GJG groups. Additionally, time to treatment failure (TTF) was calculated among patients in both groups receiving oxaliplatin-based chemotherapy as a first-line chemotherapy. Severe adverse events (evaluated as CTC grades 3 and 4) were also analyzed in both groups.

## 3. Regimen of Chemotherapy

 Patients on mFOLFOX6 received concurrent oxaliplatin (85 mg/m^2^) and leucovorin (LV) (400 mg/m^2^) for 2 h on day 1 followed by bolus 5-fluorouracil (5-FU) (400 mg/m^2^) and subsequent continuous infusion of 5-FU (2400 mg/m^2^) over 46 h. Patients on XELOX received oral capecitabine (2000 mg/m^2^/day) twice daily after breakfast and dinner from day 1 to day 14 and intravenous oxaliplatin (130 mg/m^2^) for 2 h on day 1 repeatedly every 3 weeks. Concomitant intravenous bevacizumab (7.5 mg/kg) was administered for 90 min on first administration and for 30 min on the second and all subsequent administration. Other chemotherapeutic agents were administered according to standard protocols. 

## 4. Goshajinkigan

**Figure 1:**
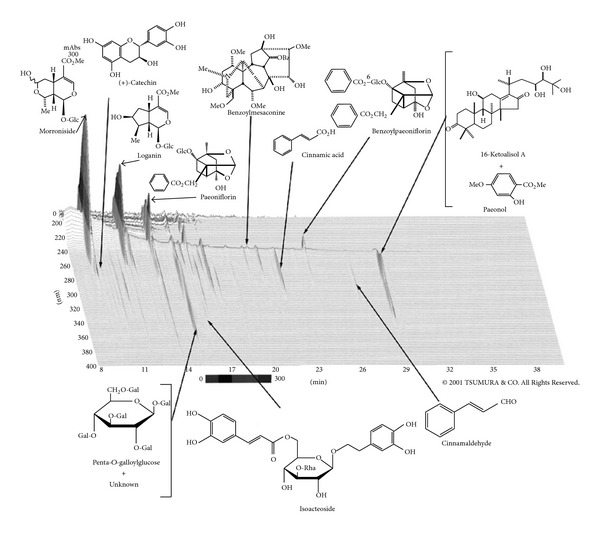
HPLC profile of Goshajinkigan.

GJG is a mixture of extracts from 10 botanical raw materials in fixed proportions: Rehmanniae Radix (Scrophulariaceae, prepared rehmanniae root, 5.0 g), Achyranthis Radix (Amaranthaceae, *Achyranthes* root, 3.0 g), Corni Fructus (Cornaceae, cornus fruit, 3.0 g), Dioscoreae Rhizoma (Dioscoreaceae, Chinese yam, 3.0 g), Plantaginis Semen (Plantaginaceae, *Plantago* seeds, 3.0 g), Alismatis Rhizoma (Alismataceae, *Alisma* rhizome, 3.0 g), *Poria cocos* (Polyporaceae, hoelen, 3.0 g), Moutan Cortex (Ranunculaceae, mountain root bark, 3.0 g), Cinnamon Cortex (Lauraceae, cinnamon bark, 1.0 g), and Aconiti Tuber (Ranunculaceae, prepared aconite root, 1.0 g). The 10 botanical raw materials in GJG were decocted in a 10-fold volume of purified water at 95°C for 1 h, which was then filtered. The filtrate was subsequently spray-dried to obtain extract powder (yield, 16.1%). For the analysis of GJG components, 1.0 g of GJG was ultrasonicated in 20 mL of methanol for 30 min and centrifuged at 3000 rpm for 5 min. The supernatant was filtered through a 0.45 *μ*m membrane and used for high-performance liquid chromatography (HPLC) analysis. The three-dimensional HPLC profile of GJG is shown in Figure 1. GJG extract powder (Tsumura & Co., Tokyo, Japan) was used here, and the dosing scheme consisted of 2.5 g orally 3 times daily before or between meals for a total of 7.5 g/day.

## 5. Statistical Analysis

 Statistical analyses were performed using the Mann-Whitney *U* test, chi-square test, and Kaplan-Meier analysis. Continuous variables such as patient age, oxaliplatin dose, and TTF were analyzed using the Mann-Whitney *U* test. Categorical variables such as the sex ratio, rate of FOLFOX and XELOX administration, stage, PS, effect of GJG, and adverse events were analyzed using the chi-square test. Kaplan-Meier analysis was performed to compare peripheral neuropathy to the total dose of oxaliplatin in patients treated with or without GJG. A *P* value less than 0.05 was considered statistically significant. 

## 6. Results

The characteristics of the GJG and non-GJG groups are shown in Table 1. There were no significant differences with respect to age, sex, ECOG PS, stage, or chemotherapy regimen. The cumulative dose of oxaliplatin (median ± SD, mg) was not significantly different between the two groups (1127 ± 390 and 1134 ± 487 in the GJG and non-GJG groups, resp.).

In the GJG group, the effect of GJG administration for the treatment of peripheral neuropathy was curative in 1 patient (3.4%), effective in 6 (20.7%), stabilizing in 20 (69.0%), and deleterious in 2 (6.9%) (Table 2). In the non-GJG group, the effect was curative in 2 patients (4.5%), effective in 7 (15.9%), stabilizing in 20 (45.5%), and deleterious in 15 (34.1%). There was a significant difference in the occurrence of deleterious effects between the GJG and non-GJG groups (*P* = 0.04). Subgroup analysis was performed with respect to the period of GJG administration. The effect of GJG treatment from the beginning of chemotherapy was curative in 1 patient (7.7%), effective in 2 (15.4%), stabilizing in 11 (69.2%), and deleterious in 1 (7.7%) (Table 3). The effect of GJG treatment starting from the middle of chemotherapy was effective in four patients (28.6%), stabilizing in nine (64.3%), and deleterious in one (7.1%). 

**Figure 2:**
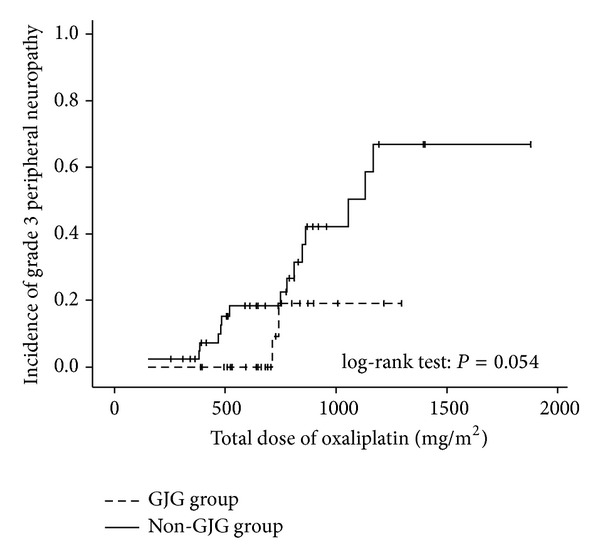
Kaplan-Meier analysis of the incidence of grade 3 peripheral neuropathy with respect to the dose of cumulative oxaliplatin in the GJG and non-GJG groups.

Kaplan-Meier analysis showed that the incidence of grade 3 peripheral neuropathy in the GJG group was lower than that in the non-GJG group. However, there was no significant difference between the groups (*P* = 0.054) (Figure 2). 

CTC grade 3 and 4 adverse events that occurred in the GJG group are described in Table 4. With the exception of peripheral neuropathy, there were no significant differences in grade 3 and 4 adverse events between the GJG and non-GJG groups. 

 The TTFs in the GJG and non-GJG groups were 7.2 and 7.6, respectively (Table 5). There were no significant differences between the groups.

## 7. Discussion

The current study revealed the efficacy of GJG administration for the treatment of peripheral neuropathy due to oxaliplatin-based chemotherapy. Four or more weeks of GJG administration enabled us to prevent grade 3 peripheral neuropathy. 

Oxaliplatin-induced peripheral neuropathy sometimes results in the discontinuation of chemotherapy. Tournigand et al. developed a stop-and-go dosing scheme using FOLFOX7 including oxaliplatin, that represented a seminal approach for the prevention of severe peripheral neuropathy [20]. This strategy used 5-FU-LV and withheld oxaliplatin for six months. The aim of this method was to reduce the severity of neuropathic symptoms such that the cumulative oxaliplatin dose could be increased. The stop-and-go method appears to be a promising approach that does not compromise the efficacy of platinate therapy and mitigates the severity of neuropathy. Another attempt to prevent peripheral neuropathy involved intravenous calcium and magnesium (Ca/Mg) infusions. In a retrospective study, 96 patients received 1 g each of calcium gluconate and magnesium sulfate intravenously before and after 3 different protocols of oxaliplatin and 5FU-LV [21]. The results showed a significantly lower incidence of peripheral neuropathy in the Ca/Mg group (27%) versus the non-Ca/Mg group (75%). Additionally, the use of glutathione and carbamazepine for the prevention of peripheral neuropathy in single-arm studies appears promising [22, 23], and a retrospective multivariate analysis of various factors has identified NSAIDS as potentially useful for neuropathic pain [24]. Drugs like pregabalin and opioids may be effective in improving neuropathic pain [6]; however, they require special precautions due to unwanted adverse effects such as sleepiness and dizziness.

Kono et al. indicated the efficacy of prophylactic GJG administration for oxaliplatin-induced neuropathy [15]. In their retrospective study, 90 patients with colorectal cancer receiving either FOLFOX4 or mFOLFOX6 were analyzed. Patients were divided into four groups based on peripheral neuropathy treatment: groups A, B, C, and D received GJG, Ca/Mg, GJG with Ca/Mg, and no treatment, respectively. The incidence and severity of peripheral neuropathy were evaluated among these groups. When the cumulative dose of oxaliplatin exceeded 700 mg/m^2^, the incidence of grade 3 peripheral neuropathy was 0% in groups A and C that received GJG. However, the incidence was approximately 20–30% in groups B and D that did not receive GJG. Thus, the incidence of grade 3 peripheral neuropathy was prevented by the administration of GJG. Additionally, this study showed that GJG prevented the occurrence of grade 1 peripheral neuropathy. However, in our study, the evaluations such as “curative” and “effective” indicated grade 0 and grade 1 neuropathy, respectively, and the ratios of these two evaluations in the GJG group were not significantly different with those in the non-GJG group. Thus, we thought the effectiveness of GJG was the reduction of only grade III neuropathy. This should be proved in further large scale trial. Nishioka et al. also reported the efficacy of prophylactic GJG administration for the treatment of peripheral neuropathy due to oxaliplatin-based chemotherapies such as mFOLFOX6 and revealed that the incidence of grade 3 peripheral neuropathy in the GJG and non-GJG groups was 0% and 12%, respectively, after 10 cycles and 33% and 75%, respectively, after 20 cycles [16]. This suggested that GJG helps suppress the onset of severe neuropathic symptoms. In our study, the incidence of grade 3 peripheral neuropathy was 6.9% in the GJG group and 34.0% in the non-GJG group. Thus, GJG enabled us to prevent grade 3 peripheral neuropathy. However our study was retrospective setting, and patients' condition in the GJG group was well balanced in some characteristics about PS and patients ages, unintentionally. Thus, we thought of the possibility that GJG might prevent the grade 3 peripheral neuropathy especially in well balanced condition patients. Additionally, Kaplan-Meier analysis indicated that the incidence of grade 3 peripheral neuropathy in the GJG group was not significantly lower than that in the non-GJG group (*P* = 0.054). A possible reason for this is that only a small number of patients were evaluated. These should be proved in future prospective trials. 

Previous studies about oxaliplatin-induced neuropathy only showed the effectiveness of prophylactic GJG administration. On the other hand, our study included patients receiving GJG from the middle of chemotherapy, and the effect of GJG on the treatment of peripheral neuropathy was effective, stabilizing, and deleterious in 28.6%, 64.3%, and 7.1% of these patients, respectively. Thus, our study revealed the potential of GJG not only as a prophylactic agent for peripheral neuropathy but also as a therapy. Regarding the timing of GJG, Kaku et al. also reported that the administration of GJG after the incidence of peripheral neuropathy was effective in patients receiving paclitaxel/carboplatin chemotherapy and indicated that CTCAE grade 3 neurotoxicity developed less frequently in patients who received GJG for 6 weeks after the incidence of peripheral neuropathy (0%) compared with patients who did not receive GJG (14.3%) [25].

De Gramont et al. reported the incidence of peripheral neuropathy in patients undergoing oxaliplatin-based chemotherapy in Western countries [26]. The authors showed that functional impairment occurred in 10% of patients at a total oxaliplatin dose of 850 mg/m^2^, and this increased to 25% at a total dose of 1020 mg/m^2^. In our study, the incidence of grade 3 peripheral neuropathy without GJG administration was higher than that reported by de Gramont et al. while other Japanese studies (described above) showed the incidence similar to ours. One possible reason for this discrepancy is that Japanese people are more susceptible to neuropathic injury due to oxaliplatin than European people; another possible reason is that the evaluation of peripheral neuropathy was subjective in our study. Further objective evaluation will be required to clarify these issues. 

Adverse events relating to GJG were analyzed in several studies [15, 16]. There were no significant differences regarding serious adverse events between the patients receiving GJG and controls. Similarly, the frequency of severe adverse events in the GJG group was similar to that in the non-GJG group in our study. About the efficacy of chemotherapy, Nishioka et al. reported that the tumor response of GJG and non-GJG patients was 68% and 57%, respectively, suggesting that GJG had no bearing on treatment efficacy. Kono et al. reported that the rates of tumor response were similar in patients with GJG and patients without GJG [15]. These authors also reported that there were no significant differences in TTF between patients receiving GJG and those not receiving GJG. Similarly, our study showed that there were no significant differences in TTF between the GJG group and the non-GJG group (Table 5). Hosokawa et al. showed that GJG did not affect the antitumor activity of oxaliplatin in rats [13]. However, details regarding the pharmacokinetics and interactions of GJG remain unknown. Further experimental studies will be required in the near future.

We also analyzed the efficacy of GJG administration with respect to the duration of GJG in colorectal cancer patients receiving oxaliplatin-based chemotherapy (Table 6). Previous studies about oxaliplatin-induced neuropathy did not show the details about the duration of GJG administration. The taste of GJG is unique and bitter. Thus, continuing GJG administration is difficult in some patients. It is important to analyze the minimum duration of GJG to achieve the effectiveness of GJG. In patients receiving GJG for less than three weeks for the treatment of peripheral neuropathy, the effect was curative, effective, stabilizing, and deleterious in 0%, 12.5%, 62.5%, and 25.0% of the patients. This shows that the duration of GJG administration should be ≥4 weeks. On the other hand, continuing peripheral neuropathy after oxaliplatin-based chemotherapy is known to occur. In some patients, symptoms worsen until two to three months after chemotherapy. André et al. reported that 60% of patients remained symptomatic after a month while 40% were symptomatic after six months [27]. The administration of GJG may improve this symptom and reduce the duration of continuing peripheral neuropathy. 

 In the current study, we concluded that GJG administration for peripheral neuropathy due to oxaliplatin-based chemotherapy was effective. The administration of GJG for 4 weeks or more, both from the beginning of chemotherapy and from the middle of chemotherapy, helps prevent grade 3 peripheral neuropathy.

## 8. Study Limitations

This study was a retrospective review of patient medical records. Clinical doctors decided whether GJG was to be prescribed from the beginning of chemotherapy. Additionally, patients were placed into the GJG group or non-GJG group according to the clinical doctor's decision. The small number of patients (29 patients) in the GJG group was not balanced with the non-GJG group (44 patients). Thus, the selection could have been biased. Analyses of the grade of peripheral neuropathy and treatment effects of GJG were based on patient interviews with the help of outpatient-center nurses and were therefore subjective. 

## Figures and Tables

**Table 1:** Characteristics of patients receiving Goshajinkigan for peripheral neuropathy due to oxaliplatin-based chemotherapy for colorectal cancer.

	GJG	Non-GJG	*P*
Number	29	44	
Age	60.4 ± 9.9	64.5 ± 11.6	N.S.
Sex (M/F)	17/12	26/18	N.S.
ECOG PS 0-1/2	25/4	40/4	N.S.
Ratio of stage 3/stage 4	7/22	13/31	N.S.
Ratio of FOLFOX/XELOX	17/12	25/19	N.S.
Median cumulative dose of oxaliplatin (mg)	1127 ± 390	1134 ± 487	N.S.

PS: performance status; GJG: Goshajinkigan; *P*: *P* value; N.S.: not significant.

**Table 2:** Effectiveness of Goshajinkigan for treating peripheral neuropathy during oxaliplatin-based treatments for colorectal cancer.

	GJG *N* = 29	Non-GJG *N* = 44	*P*
Effect on peripheral neuropathy	Curative 1/29, 3.4%	Curative 2/44, 4.5%	*vs.** *P* = 0.04
	Effective 6/29, 20.7%	Effective 7/44, 15.9%	
	Stabilizing 20/29, 69.0%	Stabilizing 20/44, 45.5%	
	Deleterious 2/29, 6.9%*	Deleterious 15/44, 34.1%**	

GJG: Goshajinkigan; *P*: *P* value.

**Table 3:** Subgroup analysis of the period of Goshajinkigan administration for peripheral neuropathy.

	From the beginning of chemotherapy *N* = 15	From the middle of chemotherapy *N* = 14
Effect on peripheral neuropathy	Curative 1/15, 7.7%	Curative 0/14, 0%
	Effective 2/15, 15.4%	Effective 4/14, 28.6%
	Stabilizing 11/15, 69.2%	Stabilizing 9/14, 64.3%
	Deleterious 1/15, 7.7%	Deleterious 1/14, 7.1%

**Table 4:** Adverse events of grades 3 and 4 observed in the GJG and non-GJG groups receiving oxaliplatin-based chemotherapy.

	GJG group *N* = 29	Non-GJG group *N* = 44
General fatigue	0	4/44, 9.0%
Allergic reaction	0	1/44, 2.2%
Oral mucositis	2/29, 6.9%	1/44, 2.2%
Disorder of blood	3/29, 10.3%	4/44, 9.0%
Infection	1/29, 3.4%	4/44, 9.0%
Nausea and vomiting	4/29, 13.7%	7/44, 15.9%
Diarrhea	2/29, 6.9%	3/44, 6.8%
Hand-foot syndrome	0	1/44, 2.2%
Pain	1/29, 3.4%	2/44, 4.5%
Peripheral neuropathy	2/29, 6.9%	15/44, 34.0%

**Table 5:** Time to treatment failure in patients receiving oxaliplatin-based chemotherapy with or without Goshajinkigan as a first-line treatment. Values are given as the median (months).

	GJG *N* = 23	Non-GJG *N* = 23	*P*
TTF	7.2	7.6	N.S.

GJG: Goshajinkigan; TTF: time to treatment failure; *P*: *P* value.

**Table 6:** Efficacy of Goshajinkigan for treatment of peripheral neuropathy with respect to duration of Goshajinkigan administration.

	GJG ≤ 3 weeks *N* = 8	GJG ≥ 4 weeks *N* = 29
Effect on peripheral neuropathy	Curative 0/8, 0%	Curative 1/29, 3.4%
	Effective 1/8, 12.5%	Effective 6/29, 20.7%
	Stabilizing 5/8, 62.5%	Stabilizing 20/29, 69.0%
	Deleterious 2/8, 25.0%	Deleterious 2/29, 6.9%
